# Implementing exercise in cancer care: study protocol to evaluate a community-based exercise program for people with cancer

**DOI:** 10.1186/s12885-017-3092-0

**Published:** 2017-02-06

**Authors:** Prue Cormie, Stephanie Lamb, Robert U. Newton, Lani Valentine, Sandy McKiernan, Nigel Spry, David Joseph, Dennis R. Taaffe, Christopher M. Doran, Daniel A. Galvão

**Affiliations:** 1 Institute for Health and Ageing, Australian Catholic University, Level 6, 215 Spring Street, Melbourne, VIC 3000 Australia; 2 Exercise Medicine Research Institute, Edith Cowan University, Perth, WA Australia; 3 Cancer Council Western Australia, Perth, WA Australia; 4 University of Queensland Centre for Clinical Research, Brisbane, QLD Australia; 5 Cancer Centre, Sir Charles Gairdner Hospital, Perth, WA Australia; 6 Faculty of Medicine, University of Western Australia, Perth, WA Australia; 7 School of Medicine, University of Wollongong, Wollongong, NSW Australia; 8 School Human Health and Social Sciences, Central Queensland University, Brisbane, QLD Australia

**Keywords:** Exercise, Physical activity, Cancer survivorship, Supportive care, Translation

## Abstract

**Background:**

Clinical research has established the efficacy of exercise in reducing treatment-related side-effects and increasing wellbeing in people with cancer. Major oncology organisations have identified the importance of incorporating exercise in comprehensive cancer care but information regarding effective approaches to translating evidence into practice is lacking. This paper describes the implementation of a community-based exercise program for people with cancer and the protocol for program evaluation.

**Methods/Design:**

The Life Now Exercise program is a community-based exercise intervention designed to mitigate and rehabilitate the adverse effects of cancer and its treatment and improve physical and psychosocial wellbeing in people with cancer. Involvement in the program is open to people with any diagnosis of cancer who are currently receiving treatment or within 2 years of completing treatment. The 3-month intervention consists of twice weekly group-based exercise sessions administered in community exercise clinics under the supervision of exercise physiologists trained to deliver the program. Evaluation of the program involves measures of uptake, safety, adherence and effectiveness (including cost effectiveness) as assessed at the completion of the program and 6 months follow-up.

**Discussion:**

To bridge the gap between research and practice, the Life Now Exercise program was designed and implemented to provide people with cancer access to evidence-based exercise medicine. The framework for program implementation and evaluation offers insight into the development of feasible, generalizable and sustainable supportive care services involving exercise. Community-based exercise programs specifically designed for people with cancer are necessary to facilitate adherence to international guidelines advising patients to participate in high-quality exercise.

**Trial Registration:**

ACTRN12616001669482 (retrospectively registered 5 Dec 2016).

## Background

Cancer is a leading cause of disease burden worldwide [1]. The combination of increasing cancer prevalence and survival rates has led to a large and rapidly growing population with unique health care requirements [2]. People with cancer experience serious chronic health sequelae most commonly fatigue, accelerated functional decline, pain, psychological distress and a higher risk of developing comorbid conditions such as cardiovascular disease, diabetes, osteoporosis and sarcopenia [3–5]. As a consequence, people with cancer experience considerable morbidity, reduced quality of life and a greater risk of losing independence as they age, which leads to increased economic burden on health care systems [6]. The observation of significantly higher primary health care use in people with cancer 2–5 years post diagnosis compared to age-matched controls supports this contention [7]. While advances have been made in care, current medical and allied health care services are inadequate to address the demand for the management of chronic and late-appearing effects of cancer and its treatment [8].

Epidemiological, clinical and laboratory-based research has established appropriate exercise as a safe and effective medicine for people with cancer which results in improved disease, physical and psychological outcomes [9–11]. For example, appropriate exercise prescription has been shown to improve quality of life across multiple general health and cancer-specific domains, reduce cancer-related fatigue, alleviate psychological distress and counteract functional declines [11–14]. The increasing body of evidence has led major health organisations (e.g., American Cancer Society, National Comprehensive Cancer Network) to recommend exercise as essential for people with cancer [15–17]. Despite these recommendations which are disseminated by government and non-government cancer organisations worldwide, the majority of people with cancer do not participate in appropriate levels of exercise [18–20]. Approximately 50–70% of people with cancer do not meet weekly recommendations of at least 150 m of moderately intense aerobic exercise [18–21]. While guidelines recommend moderate intensity muscle strengthening exercises involving all major muscle groups to be performed at least two times per week, data in adults without cancer suggest that only ~15% of adults ≥45 years meet resistance exercise guidelines [21–23]. Minimal information currently exists regarding the prevalence of resistance exercise amongst people with cancer, however, a recent report suggests approximately 12% of men with prostate cancer met the resistance exercise guidelines [21]. People with cancer have indicated a willingness and desire to participate in appropriately designed and delivered exercise programs [24, 25]. However, inactivity data relating to both aerobic and resistance exercise modalities indicates that current supportive care services are ineffective in providing access to appropriate exercise programs for people with cancer and promoting long-term exercise adherence [18–23].

Despite the established benefits of exercise for people with cancer and calls to include exercise as a component of comprehensive cancer care [26], translation strategies for the integration of efficacious exercise programs into routine cancer care are limited. There is a clear paucity of research investigating the design and implementation of exercise programs that are accessible and generalizable to a large proportion of people with cancer (i.e., administered in a standard supportive care setting). The purpose of this paper is to describe the implementation of a community-based exercise program for people with cancer and the protocol for program evaluation.

## Methods/Design

An effectiveness/pragmatic study design was applied to examine the implementation of an exercise program for people with cancer in a real-world, standard practice setting [27]. This approach was adopted to account for external patient-, health professional- and health system-factors that may influence the magnitude of effect observed when exercise interventions are delivered in standard practice settings (i.e., not highly controlled research trials). Thus, to ensure high external validity, broad inclusion criteria and few exclusion criteria were applied and the exercise intervention was delivered in circumstances that reflect routine practice.

### Program design

The Life Now Exercise program is a community-based exercise intervention designed to mitigate and rehabilitate the adverse effects of cancer and its treatment and improve physical and psychosocial wellbeing in people with cancer. The mandate of the program is to provide people with cancer access to a cancer-specific exercise program delivered using evidence-based practice. An additional goal of the program is building capacity of exercise physiologists to provide best practice exercise prescription and supervision to people with cancer. Implementation of the program is driven by international guidelines recommending high quality exercise for all people with cancer and the failure of existing resources to engage patients in such behaviour.

The program is administered throughout Western Australia by Cancer Council Western Australia, a state based not-for-profit cancer organisation. Community donations provided to the organisation funds the program which is subsidised for participants so that they can complete the 3-month intervention at no personal financial cost. The program is delivered at a range of community based exercise clinics (typically ~10 per year) that span metropolitan and regional areas of Western Australia. The program operates over three terms per year, catering for up to 150 participants each term (typically ~80–120 participants/term).

**Fig. 1:**
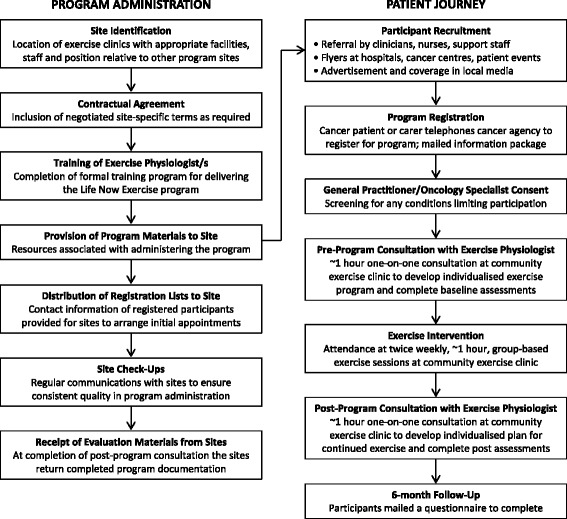
Process for program implementation and evaluation

A series of elements were developed to support the implementation of the Life Now Exercise program. These include processes for identifying suitable community-based sites, establishing formal agreements with each site, training and supporting exercise physiologists to deliver the program as designed, engaging people with cancer to participate in the program, screening participants to ensure their health status is adequate to exercise safely in the program environment, and program evaluation (Fig. 1). This framework was developed to facilitate sustainable adoption and maximise generalizability beyond a single state-based supportive care program.

### Participants

People with any diagnosis of cancer who are currently receiving treatment or within 2 years of completing treatment for cancer are eligible to participate. Exclusion criteria are applied to minimise risk of harm associated with program participation, these are: 1) neutropenia; 2) severe anaemia; 3) bone metastases; or 4) any musculoskeletal, cardiovascular or neurological disorder that could place the participant at risk of injury or illness resulting from the exercise program (as determined by the patient’s physician). No age restrictions are applied but people with cancer are required to obtain physician consent prior to participating in the Life Now Exercise program. Carers of eligible participants are permitted to concurrently attend the program.

Potential participants are required to self-enrol in the Life Now Exercise program by telephoning the Cancer Council Western Australia. Multiple approaches are adopted to raise awareness of the program including: education for oncology clinicians and support staff to facilitate direct referral of patients; distribution of program flyers at hospitals, cancer centres and community-based organisations as well as health professional and patient events; email and mail out communications to people who have contacted Cancer Council WA and expressed interest in exercise; advertisement and coverage in local media; and information provided on the Cancer Council Western Australia website and social media accounts. People with cancer who register are mailed an information package containing resources, screening documentation and contact details of the relevant program site. If the number of registrants exceeds the capacity of a site, participants are placed on a wait list for the next term of the program.

The program evaluation protocol was approved by the Edith Cowan University Human Research Ethics Committee (ID: 6192) and all participants involved in the evaluation provided written informed consent.

### Exercise intervention

The Life Now Exercise program intervention was designed in accordance with international guidelines for best practice exercise prescription for people with cancer [15–17]. In order to facilitate widespread participation of people with cancer, specific consideration was given to the balance between maximising: 1) physiological and psychosocial benefits; 2) accessibility of the program; and 3) long-term feasibility of the program. As such, the intervention consists of a pre- and post- program individual exercise physiologist consultation and 3 months of twice weekly group-based exercise sessions administered in community exercise clinics under the supervision of exercise physiologists specifically trained to deliver the Life Now Exercise program. No formal intervention was provided after the completion of the 3-month Life Now Exercise program however, participants could continue to access the same exercise clinic/fitness centre/gym on an ongoing basis using the standard user-pays model.

#### Individual consultations

Prior to commencing the program, each participant receives a one-on-one consultation with the exercise physiologist lasting approximately 1 h in duration. This consultation involves screening of health status and initial assessment in order to individualise the exercise prescription to their specific needs according to their cancer site, stage and treatment history, severity of any symptoms/side effects, as well as general health history, physical abilities and personal preferences. Each participant’s exercise prescription is designed to provide optimal stimulus to the cardiorespiratory and neuromuscular systems while maximising safety, compliance and retention. Following completion of the program, participants receive a second individual consultation. The intention of this service is to conduct assessments and report progress since initiating the program, discuss strategies to continue exercising after the program and develop a plan to maintain positive exercise behaviour long-term. The cost of these consultations is subsidised through the Australian public health system (Medicare) Chronic Disease Management plan.

#### Group exercise sessions

Twice weekly exercise sessions are conducted in groups of approximately 10 participants under the supervision of an accredited exercise physiologist. The maximum number of participants per group is 15–20 (site dependent) and the groups are administered with an exercise physiologist to participant ratio of up to 1:10. These sessions are delivered in community based exercise clinics, fitness centres and gym facilities. The sessions last approximately 1 h in duration and consist of a combination of moderate to high intensity aerobic and resistance exercise as well as flexibility exercises. The aerobic exercise component includes 20 to 30 min of at least moderate intensity cardiovascular exercise using a variety of modes such as walking or jogging on a treadmill, cycling or rowing on a stationary ergometer. The target intensity is between 60 to 85% of estimated heart rate maximum. The resistance exercise component involves 6 to 10 exercises that target the major upper and lower body muscle groups (e.g., leg press, leg extension, leg curl, calf raise, chest press, lat pulldown, bicep curl, tricep extension). Target intensity is manipulated from 6 to 12 repetition maximum (RM; i.e., the maximal weight that can be lifted 6 to 12 times which is equivalent to ~60–85% of 1RM) using 1–4 sets per exercise. Resistance exercise selection is individually prescribed. The flexibility component involves approximately 5 min of stretching of the major muscle groups for 15–30 s duration each. Exercise prescription is progressive and modified according to individual response. Session ratings of perceived exertion (RPE) are recorded after the completion of each exercise session to monitor the perceived intensity of the exercise using the 6–20 Borg scale [28]. The target session RPE range is 12–16 [15]. Participants are encouraged to undertake additional home-based aerobic exercise with the goal of achieving a total of at least 150 min of moderate intensity aerobic exercise each week (accumulated through the combination of clinic- and home-based sessions). Participants are provided with a logbook to help monitor their home-based exercise levels but these data are not collected or analysed.

#### Exercise physiologist training program

Exercise physiologists delivering the Life Now Exercise program undergo a formal training course titled the Life Now Instructor Course. The course is designed to provide the knowledge and skills required to apply evidence based practice for people with cancer. The course involves a theory component consisting of approximately 10 h of online course material and a practical component consisting of an 8-h workshop. The Life Now Instructor Course is an accredited continuing education program with Exercise and Sports Science Australia which is Australia’s peak professional body for exercise science. Of note, accredited exercise physiologists in Australia are required to complete a 4 year tertiary degree in clinical exercise physiology and maintain accreditation through meeting annual professional development requirements. Only accredited exercise physiologists have the provider status to enable Medicare rebate for the individual consultations involved with the Life Now Exercise program.

#### Behaviour change theory

The exercise intervention is theoretically underpinned by the Theory of Planned Behaviour [29], the most widely used theory of exercise motivation for people with cancer [30]. As such, in addition to technical exercise instruction, exercise physiologists provide education and advice designed to change attitudes towards exercise (i.e., instrumental attitude, perceived benefits of performing exercise) and modify exercise beliefs (i.e., control beliefs, perceived factors that facilitate exercise behaviour) through increasing knowledge, promoting self-efficacy and assisting participants overcome barriers to exercise. This education is delivered throughout the individual consultations and group-based exercise sessions. Specific cancer education seminars are regularly delivered alongside the Life Now program by Cancer Council Western Australia but these sessions aren’t included as part of the program (i.e., isn’t a requirement for participants to attend).

### Evaluation

Evaluation of the Life Now Exercise program involves measures of uptake, safety, adherence and effectiveness of the program. These analyses incorporate elements of the RE-AIM (reach, effectiveness, adoption, implementation and maintenance) framework [31]. Evaluation of the effectiveness of the program involves comparisons among pre-program, post-program and 6 months follow-up assessments. The evaluation will be undertaken on a sample of 600 people with cancer participating in the program (refer sample size calculations below) with data collection initiating in 2011 and proceeding until the target sample size is achieved.

#### Uptake

The proportion of people who participate in the Life Now Exercise program from those eligible people with cancer in Western Australia will be reported as the participation rate. People with cancer who register for the program but do not commence participation in the program will also be reported. The representativeness of participants will be determined by comparing demographic and clinical characteristics to people diagnosed with cancer in Western Australia. Information about cancer diagnoses will be derived from the Western Australia Cancer Registry (Department of Health, Government of Western Australia; www.health.wa.gov.au/wacr).

#### Safety

The incidence and severity of any adverse events (e.g., fall, muscle strain) that occur during the clinic based sessions is monitored and reported by the supervising exercise physiologist using program specific documentation. Additionally, participants self-report incidence and severity of any adverse events they experience during the clinic- and home-based exercise using program specific documentation.

#### Adherence

Attendance at clinic-based exercise sessions and the reason for any missed sessions is tracked throughout the program. Completion of assessments at pre- and post-program time points as well as 6 months follow-up questionnaires will be reported. Compliance to the Life Now Exercise program procedures by exercise physiologists at each site is monitored through evaluation of program documentation (e.g., screening, assessment and exercise prescription documents).

#### Effectiveness-objective assessments

Objective measurements of physical function, resting blood pressure, height, weight, waist and hip circumferences occurs for all participants at pre- and post-program time points. Physical function is assessed using the 400 metre walk and repeated chair rise tests with lower time taken to complete the tests representing higher functional performance. As a measure of cardiovascular fitness, peak oxygen consumption (VO_2_peak) is estimated from the 400 metre walk test time and heart rate response [32]. A validated oscillometric device or sphygmomanometer is used to record resting brachial blood pressure. Circumferences are measured using a constant tension anthropometric tape in accordance to standard protocols. These assessments are performed in triplicate with the exception of the 400 metre walk test. These assessments are conducted by the same exercise physiologist administering the exercise intervention.

A sub-set of participants willing to attend a tertiary assessment centre complete additional objective assessments to evaluate further components of functional capacity [33]. Involvement in the additional assessments is open to any participant willing to attend an additional testing session and as such, evaluation of these endpoints will be exploratory. Maximal strength of the lower and upper body is determined using the 1RM in the leg press, chest press and seated row exercises. Usual and fast pace 6 metre walks evaluate ambulatory ability while the 6 metre backwards walk is used to assess dynamic balance. Static balance is determined using the sensory organisation test performed on a Neurocom Smart Balancemaster (Neurocom, OR, USA). Body composition and bone health are derived from dual-energy X-ray absorptiometry (DXA; Hologic Discovery A, MA, USA). Regional and whole body lean mass and fat mass as well as trunk adiposity, visceral fat and adipose indices are assessed using whole body DXA scans. Areal bone mineral density of the hip (total hip) and lumbar spine (L_2–4_) as well as whole body bone mineral content is measured by DXA using standard procedures. These assessments are conducted by an independent research assistant not involved with administering the exercise intervention.

#### Effectiveness-patient reported outcomes

A series of questionnaires with sound psychometric properties are utilised to assess general health and cancer specific quality of life, cancer-related fatigue, psychological distress, and exercise behaviour and motivation. Evaluation of patient reported outcomes occurs across all time points. The Medical Outcomes Study 36-Item Short-Form Health Survey (SF-36) is used to assess general health-related quality of life status across physical functioning, physical role functioning, bodily pain, general health, vitality, social functioning, emotional role functioning and mental health domains (higher scores represent greater quality of life) [34]. Cancer specific quality of life is evaluated by the European Organisation for Research and Treatment of Cancer (QLQ-C-30) questionnaire [35]. The QLQ-C-30 questionnaire includes five functional domains (physical, role, cognitive, emotional and social; higher scores represent greater function/quality of life) and three symptom scales (fatigue, pain and nausea; lower scores represent greater quality of life/less symptom severity). Cancer-related fatigue is assessed using the Functional Assessment of Chronic Illness Therapy-Fatigue (FACIT-Fatigue) scale (higher scores indicate less fatigue) [36]. The Brief Symptom Inventory-18 (BSI-18) is used to evaluate psychological distress across the domains of depression, anxiety, somatization and global distress severity (lower scores represent less distress) [37]. Self-reported exercise levels are assessed by the Godin Leisure-Time Exercise Questionnaire modified to include participation in resistance exercise [38]. Determinants of exercise motivation are derived from the Theory of Planned Behaviour and assessed using validated questionnaires. The Theory of Planned Behaviour constructs (attitude, subjective norm, perceived behavioural control and intention) are assessed in accordance with established guidelines [29]. Participants complete these questionnaires independently at a location of their selection outside the exercise facility they attend the Life Now Exercise program at.

#### Cost effectiveness

Cost-effectiveness of the Life Now Exercise program will be evaluated using the ACE-Prevention methodology. These methods are international best-practice for cost-effectiveness analyses in health care and include: adoption of a social perspective; transparent and scientific methods to identify, measure and value both costs and outcomes from the trial; modelling and uncertainty testing of epidemiological and costing input parameters; and, interpretation of results within a broader decision-making framework [39]. The cost-effectiveness analysis will model costs and outcomes for the duration of the trial and for a 10-year period, discounting future costs and health outcomes at a rate of 3% per year. The costs and health outcomes will be summed to determine the incremental cost-effectiveness ratio (ICER) in dollars per quality adjusted life years (QALY) gained. QALYs will be derived from the SF-6D utility index score obtained from the SF-36 using standard methods [40]. Monte Carlo analysis will be used to derive 95% uncertainty intervals for all outcomes and to determine the probability of intervention cost-effectiveness against a cost-effectiveness threshold of $50,000 per QALY. ICER results will be displayed on a cost-effectiveness plane with affordability issues addressed in an acceptability curve. The results of the cost-effectiveness analysis will be considered in the context of other decision making criteria including: strength of evidence; capacity of the intervention to reduce inequity; acceptability to stakeholders; feasibility; sustainability; and potential for other consequences.

#### Statistical analyses

Data will be analysed using an intention-to-treat approach with maximum likelihood imputation of missing values. Analyses will include standard descriptive statistics, Student’s *t*-tests, chi-square, correlation, regression and repeated measures ANOVA (or ANCOVA as appropriate) to examine differences over time. Clinically relevant covariates will be included in analyses. Sub-group analyses based on cancer site and anti-cancer treatment status will be conducted. Investigations into responders and non-responders will be conducted to explore heterogeneity of intervention effect.

Sample size calculations were based on having sufficient power to detect a small effect (*d* = 0.02) in study endpoints. Given the number of planned assessments, correction for multiple testing is required. An alpha level of 0.001 was applied which provides adjustment for up to 50 tests. A priori, 420 participants are required to achieve 80% power at an alpha level of 0.001 (two tailed) to demonstrate a mean paired difference of *d* = 0.02 from pre- to post-program. To ensure sufficient participant numbers at the completion of the Life Now Exercise program, sample size calculations accounted for a 30% attrition rate. Thus the evaluation will be conducted on a sample size of 600 participants.

## Discussion

Clinical research has established appropriate exercise as an effective adjunct therapy for people with cancer [9–11, 15], oncology organisations have identified the importance of incorporating exercise in cancer care [15–17] and people with cancer have indicated their wish to participate in appropriately designed and delivered exercise programs [24, 25]. However, information regarding effective approaches to incorporate exercise into routine care is limited. A major challenge is to translate knowledge of efficacious exercise interventions into practice with feasible, scalable and sustainable programs that are generalizable for all people with cancer. The Life Now Exercise program has been developed to bridge the gap between research and practice, and generate data to help guide implementation strategies/models for the integration of exercise in the cancer care paradigm.

Research involving people with cancer has demonstrated that motivational outcomes are strong predictors of exercise behaviour [30]. Thus the design of the Life Now Exercise program is theoretically based on the determinants of exercise motivation and behaviour among people with cancer. The most widely used theoretical model in cancer (Theory of Planned Behaviour; TPB) suggests that people with cancer will intend and be motivated to exercise when they: 1) view it positively; 2) believe that people important to them think they should exercise; and 3) believe that exercise is under their control and they are able to perform exercise [29]. Constructs of the TPB have been reported as statistically significant predictors of the intention to participate in exercise [41, 42] and to significantly predict program attendance [42], although the strength of these predictions are moderate [43]. While limited information exists about how to use these constructs to develop interventions that enhance positive exercise behaviours, current knowledge suggests that addressing patients attitudes towards exercise, their subjective norm and perceived behavioural control are critical components of an effective exercise program [30]. Additionally, social support/social connectedness provided by bringing together peers has been identified as a key determinant of adherence to exercise programs and represents another component critical to the design of effective exercise programs [30, 44]. Furthermore, investigation of barriers to exercise among people with cancer has identified a variety, most common of which are disease specific (e.g., treatment-related side effects, especially fatigue) and factors common to non-cancer adults (e.g., time constraints, distance/travel time, weather extremes). Additional barriers include issues such as lack of facilities for people with cancer and safety concerns [24, 25]. Commonly identified facilitators of exercise in people with cancer include appropriate supervision, group based but individually tailored and gradually progressed exercise prescription [24, 25, 45]. The inclusion of feedback and approval from their oncologist or general physician were factors also identified to facilitate continued exercise participation [24, 25]. Application of the TPB constructs and common exercise barriers and facilitators led to program components within the Life Now Exercise program. Specifically, requiring the endorsement of oncology specialist or general physician for participation in the intervention underscores the positive value of exercise behaviour and indicates their support for exercise (as well as screening for contraindications to exercise). Upskilling of exercise physiologists allows for targeted education of patients, the ability to tailor the exercise prescription and progression to each individual and modify the prescription to manage treatment-related side effects, all of which helps allay potential concerns about safety of the intervention. Administration of the exercise program within local community-based exercise clinics enhances patients’ perceived behavioural control and limits common barriers of distance from and travel time to exercise facilities. The positive atmosphere of this environment coupled with the group-based setting may also contribute to enhanced affective attitude and subjective norm within people with cancer. Furthermore, the social support and connectedness provided by bringing together a group of people with the shared experience of cancer may facilitate adherence to the program and continuation of positive exercise behaviours longer-term. The incorporation of program elements addressing constructs of behaviour change theory, barriers and facilitators to exercise, combined with exercise physiologists’ use of evidence based practice, is designed to maximise engagement of, and potential benefits to, people with cancer.

Information arising from the implementation of the Life Now Exercise program extends existing reports of community-based exercise programs for people with cancer designed for ongoing operation [46–49]. The ‘Livestrong at the YMCA’ program operates at over 400 sites throughout America [50]. The program involves 12 weeks of twice weekly group based exercise sessions administered at YMCA facilities by personal trainers and the benefits of participation have been reported on a sample of 187 participants [47]. The ‘FitSTEPS for Life’ program operates across various community based exercise sites (e.g., community centres, churches, health centres) in Texas, USA where people with cancer receive an individualised exercise plan and are provided access to ongoing exercise supervision [51]. Improvements in a range of quality of life domains were observed following 2 years of involvement in the program on a sample of 177 participants [46]. Cancer specific community-based exercise programs have also been developed in Canada. The ‘CanWell’ program is a 12-week exercise (two supervised sessions weekly) and education program administered by staff trained in program delivery at a YMCA facility in Ontario. Evaluation of a sample of 65 participants demonstrated improvements in quality of life and physical function at the completion of the program [49]. The BEAUTY program (*Breast* cancer patients *E*ngaging in *A*ctivity while *U*ndergoing *T*reatment) is a 12-week program involving twice weekly group based exercise sessions and biweekly education sessions delivered by certified exercise physiologists at a single tertiary exercise facility in Alberta [52]. Evaluation was undertaken on a sample of 80 patients which demonstrated safety but no clinically significant improvements following program completion (possibly associated with the low attendance rate of ~30%) [48]. These data provide promising initial evidence of the effectiveness of cancer specific exercise programs implemented in the community but significant continued effort is required to increase knowledge translation and implementation approaches. Evaluation of the Life Now Exercise program extends existing reports by assessing the effectiveness of a community-based exercise program in the largest sample of people with cancer to date (*n* = 600) using a comprehensive suite of assessments. The sample size and inclusive participant criterial allows for sub-group analyses which may provide insight into how people with different cancer types and treatment status respond to exercise. Importantly, examination of which participants do and don’t respond to exercise will provide novel information regarding demographic, clinical, motivational and other characteristics/factors that influence the response to exercise in people with cancer. Further to this, the evaluation will provide insight into what kind of participants engage in a community-based exercise program which may help inform future work to target people with cancer who require additional stimulus to engage in positive exercise behaviours. Examination of the cost-effectiveness of the program represents a unique addition to the literature and significant advance in current knowledge regarding the potential value of cancer-specific exercise interventions to the health system. Additionally, detailed reporting of the elements contributing to the design and implementation of the Life Now Exercise program may help inform the development of feasible, effective and sustainable supportive care exercise services. Collectively this information will help guide future research and translational work addressing the low levels of exercise behaviour in people with cancer.

There are important limitations to note in the design and evaluation of the Life Now Exercise program. As participants self-enrol into the program they will have a level of motivation to undertake exercise that may not be representative of all people with cancer. Integrating systematic referral of all people with cancer through oncology departments and treatment centres is not possible within the scope of this study. While program sites span metropolitan and regional areas, the program is implemented in Western Australia only and limited to the context of the Australian health care system. Program participation is funded by Cancer Council Western Australia so as to be delivered at no cost to people with cancer. These factors may limit the generalizability for implementation as an on-going program in other settings. Evaluation of the program is limited by the single-group design which precludes appraisal against a comparable sample of people with cancer. Evaluation is also limited by the reliance on community-based health professionals to administer assessments and return program documentation to the research team. Assessor bias cannot be ruled out as the same health professional who delivered the intervention administered the objective assessments (note: patient reported outcomes were conducted independently by the participant). The potential influence of social support on exercise motivation and behaviour may not be adequately assessed by domains of the Theory of Planned Behaviour questionnaire. These limitations are offset by strengths to the implementation and evaluation of the Life Now Exercise program. The program incorporates theory based behaviour change strategies and applies evidence based practice in the delivery of exercise to people with cancer. External validity of the program is supported by the implementation within a community-based setting to people with any diagnosis of cancer. This is further enhanced by administering the program as a “real-world” intervention delivered in a standard supportive care service setting. Evaluation of the program is guided by the RE-AIM framework and includes a robust suite of endpoints.

There is a dearth of knowledge regarding effective approaches of translating exercise oncology evidence into cancer care. To bridge the gap between research and practice, the Life Now Exercise program was designed and implemented to provide people with cancer access to evidence based exercise medicine. The framework for program implementation and evaluation offers insight into the development of feasible, sustainable and potentially effective supportive care services involving exercise. Effective community-based exercise programs specifically designed for people with cancer may help reduce the disease burden of cancer and improve the health and wellbeing of people with cancer through increased adherence with exercise guidelines.

## Abbreviations

BSI-18: Brief Symptom Inventory-18
DXA dual: energy X-ray absorptiometry
FACIT-Fatigue: Functional Assessment of Chronic Illness Therapy-Fatigue
ICER: Incremental cost-effectiveness ratio
QALY: quality adjusted life years
QLQ-C-30: European Organisation for Research and Treatment of Cancer
RE-AIM: Reach, effectiveness, adoption, implementation and maintenance
RM: Repetition maximum
RPE: Ratings of perceived exertion
SF-36: Medical Outcomes Study 36-Item Short-Form Health Survey
TPB: Theory of Planned Behaviour
VO_2_peak: Peak oxygen consumption
YMCA: Young Men’s Christian Association
